# Effect of prevention measures on incidence of human listeriosis, France, 1987-1997.

**DOI:** 10.3201/eid0706.010610

**Published:** 2001

**Authors:** V Goulet, H de Valk, O Pierre, F Stainer, J Rocourt, V Vaillant, C Jacquet, J C Desenclos

**Affiliations:** Department of Infectious Diseases, Institut de Veille Sanitaire, Saint-Maurice, France. v.goulet@invs.sante.fr

## Abstract

To assess the impact of preventive measures by the food industry, we analyzed food monitoring data as well as trends in the incidence of listeriosis estimated through three independent sources: the National Reference Center of Listeriosis; a laboratory-based active surveillance network; and two consecutive nationwide surveys of public hospital laboratories. From 1987 to 1997, the incidence of listeriosis decreased by an estimated 68%. A substantial reduction in the proportion of Listeria monocytogenes-contaminated products was observed at the retail level. The temporal relationship between prevention measures by the food industry, reduction in L. monocytogenes-contaminated foodstuffs, and reduction in listeriosis incidence suggests a causal relationship and indicates that a substantial part of the reduction in illness is related to prevention efforts.


### Effect of Prevention Measures on Incidence of Human Listeriosis, France, 1987–1997

**Véronique Goulet,* Henriette de Valk,* Olivier Pierre,† Frédéric Stainer,‡ Jocelyne Rocourt,§ Véronique Vaillant,* Christine Jacquet,§ and Jean-Claude Desenclos***

*Institut de Veille Sanitaire, Saint-Maurice, France; †Direction Générale de la Concurrence, Bureau Hygiène, Paris, France; ‡Direction Générale de l'Alimentation, Bureau des Préventions des Contaminations, Paris, France; and §Institut Pasteur, Paris, France

To assess the impact of preventive measures by the food industry, we analyzed food monitoring data as well as trends in the incidence of listeriosis estimated through three independent sources: the National Reference Center of Listeriosis; a laboratory-based active surveillance network; and two consecutive nationwide surveys of public hospital laboratories. From 1987 to 1997, the incidence of listeriosis decreased by an estimated 68%. A substantial reduction in the proportion of *Listeria monocytogenes*-contaminated products was observed at the retail level. The temporal relationship between prevention measures by the food industry, reduction in *L. monocytogenes*-contaminated foodstuffs, and reduction in listeriosis incidence suggests a causal relationship and indicates that a substantial part of the reduction in illness is related to prevention efforts.

Although rare, invasive listeriosis is an infection of public health concern because of its severity, with a case-fatality rate evaluated at 20%-30%, the possible sequelae, and its potential to cause epidemics. In 1981, the investigation of an outbreak of listeriosis in Canada demonstrated for the first time that human listeriosis could be caused by transmission of *Listeria monocytogenes* through contaminated foods, in this case coleslaw (1). However, it was not until 1985, prompted by the outbreak of listeriosis in California linked to inadequately pasteurized soft cheese, that *L. monocytogenes* became a major concern of the food industry. In the United States, as a first step, the Food and Drug Administration began monitoring dairy products for *L. monocytogenes*. This monitoring was later expanded to include ready-to-eat foods such as cold meat and poultry products, seafood, and salads; strict zero-tolerance guidelines for *L. monocytogenes* in ready-to-eat foods were enforced (2).

In France, the first control measures for *L. monocytogenes*, including microbiologic monitoring of unprocessed and finished products, were implemented in 1986 in plants producing cheese for exportation to the United States (3). When a soft cheese was shown to be the cause of a large outbreak that occurred in Switzerland from 1983 to 1987, French authorities enforced prevention efforts to eliminate sources of contamination by *L. monocytogenes* in all cheese production plants beginning in 1988 (4). In 1992, *Listeria* control measures were extended to include plants producing ready-to-eat meat and meat products (5). In 1992, the investigation of a large outbreak (279 cases) identified pork tongue in jelly as the main vehicle of transmission and showed that cross-contamination of other ready-to-eat meat products, especially those sold at the delicatessen counter, amplified transmission (6). As a result, hygiene measures at the retail level and particularly at delicatessen counters were reinforced (7,8). This 1992 outbreak prompted the Ministry of Health to issue recommendations through the public media to immunocompromised patients, the elderly, and pregnant women to avoid certain foods, encouraging them to consult a physician in case of symptoms suggestive of listeriosis.

Finally, in 1993, *L. monocytogenes* control measures were extended to include all foods potentially contaminated with *L. monocytogenes* (9). These control measures included microbiologic monitoring of food products and, if *L. monocytogenes* were isolated, investigation, increased sanitation, and plant clean-up. Foods were considered to be potentially contaminated with *L. monocytogenes* if they were either raw and did not undergo a listericidal process (such as cooking) before being eaten, or if they were pasteurized foods that could be contaminated during handling and that have characteristics favorable to the growth of *Listeria* (e.g., pH, water content, and salt concentration within certain ranges). For some products (food for infants and toddlers, canned food) a zero-tolerance policy was enforced. For dairy, meat, and fish products, the standard is to have no contamination, but <100 CFU *L. monocytogenes*/g at the end of the product's shelf life can be tolerated under certain circumstances.

In the same period, the dairy corporation and meat processing association produced video training programs to help managers improve food hygiene understanding by educating their employees. Several guidelines were published, in 1991 and 1992 for the dairy industry and in 1994 for the meat product industry. Guidelines to introduce Hazard Analysis and Monitoring of Critical Control Points (HACCP) in manufacturing plants were promulgated in 1992 by the meat and

Address for correspondence: V. Goulet, Institut de Veille Sanitaire, 12 rue du Val d'Osne, 94415 Saint-Maurice Cedex, France; fax: 00 33 1 41 79 67 69; e-mail: v.goulet@invs.sante.fr dairy industry, and software was developed to help managers implement this method in their plants.

We analyze trends in the incidence of listeriosis to assess the impact of the measures described.

### Material and Methods

### Case Surveillance

Data on the incidence of human listeriosis in mainland France were obtained from three independent sources. The first source was the National Reference Center (NRC) of listeriosis, to which microbiology laboratories voluntarily send human isolates of *L. monocytogenes*. The NRC was the Microbiology Laboratory of the Medical Faculty in Nantes from 1982 to 1992; since 1993, the Institut Pasteur in Paris has performed this function. For patients identified by the NRC in 1997, information on pregnancy status and underlying medical conditions was obtained by contacting the attending physician (10).

The second source was two surveys carried out among public hospital laboratories to estimate listeriosis incidence in France in 1987 and 1988 (11,12). In France, general hospitals that provide primary care are distinguished from regional hospitals, usually university teaching hospitals, which provide primary as well as secondary care. With the rare exceptions of a few specialized university hospitals, all general and regional/university hospitals have neonatal or pediatric departments or both, as well as an obstetric department.

In the retrospective surveys, all laboratories of general and regional/university hospitals were asked to complete case reports for all patients from whom *L. monocytogenes* had been isolated in the year of the survey. Information was collected on the site of isolation of the strain and age and sex of the patient. In 1987, information on pregnancy status and underlying disease, as well as clinical outcome of listeriosis, was also recorded. For each year, the total number of cases in France was estimated by dividing the number of cases reported by the participating hospitals by the proportion of beds represented by these hospitals (number of beds in participating hospitals/total number of beds in all public hospitals in France).

The third source was a surveillance network of laboratories of general and regional/university hospitals called EPIBAC, which has been operational since 1987 (13). Participating laboratories complete monthly report forms indicating all cases of bacteremia or meningitis with an isolate of *L. monocytogenes*, *Streptococcus pneumoniae, Haemophilus influenzae, Neisseria meningitidis*, or *Streptococcus* groups A and B. For each case, the type of isolate and the date of birth and sex of the patient are recorded. Until 1991, a case was defined as an isolate. Therefore, a patient with isolates from both blood and cerebrospinal fluid (CSF) was reported on two separate forms as a case of bacteremia and a case of meningitis. Since 1991, cases of listeriosis are reported on a single form, and for each case it is noted whether the isolate was recovered from blood or CSF. Consequently, for the period 1987-1997, we cannot analyze trends in incidence from the total number of cases, since the number of cases before 1991 would be overestimated. Therefore, we analyzed trends in incidence from 1987 to 1997 separately from the total number of blood isolates (whether or not associated with an isolate from CSF) and from the total number of CSF isolates (whether or not associated with an isolate from blood).

For each year, the total number of cases of listeriosis in France was estimated by dividing the number of cases reported by the participating hospitals by the proportion of hospital admissions in medicine departments (all nonsurgical departments) represented by these hospitals (number of admissions in medicine departments of participating hospitals/total number of admissions in medicine departments in public hospitals in France).

### Case Definition for Surveillance

In the NRC and in the two surveys of hospital laboratories, a case of listeriosis was defined as isolation of *L. monocytogenes* from a normally sterile site or, in the case of a newborn <7 days of age, from any site. The EPIBAC network, which used a more restricted case definition, reported only invasive cases with an isolate of *L. monocytogenes* from blood or CSF. Patients with blood or CSF isolates represent approximately 80% of all cases identified by laboratory surveys and the NRC. Pregnancy-associated cases were defined by the isolation of *L. monocytogenes* from a pregnant woman or her fetus or newborn infant within the first 30 days of life. A mother-infant pair (even in the case of twins) was counted as a single case. A predisposing condition was defined as one for which an increased risk of listeriosis had been demonstrated in a previous study (14; manuscript in preparation). These conditions included malignancies, chronic hemodialysis, hepatic or renal failure, diabetes, HIV/AIDS, corticotherapy, chemotherapy, and organ transplantation.

### Sensitivity of Data Sources

The number of laboratories sending isolates to the NRC increased over time. In 1986, comparison with the number of cases identified by a survey among hospital laboratories estimated this proportion at <33% of the isolates (15). For 1997, the sensitivity for case-detection by EPIBAC and NRC was evaluated by the capture-recapture method (16). A total of 225 cases with *L. monocytogenenes* isolated from blood or CSF were identified by one or both systems: 148 by EPIBAC, 190 by NRC, and 113 by both systems. The sensitivity for detecting cases of bacteremia or meningitis was estimated at 76% (95% confidence interval [CI] 72-81) for NRC and 59% (95% CI 56-63) for EPIBAC. The annual incidence of invasive listeriosis in 1997 was thus estimated for NRC by dividing the number of identified cases by 76%.

### Food Monitoring

In France, two ministries are responsible for monitoring foods for *L. monocytogenes*. The Ministry of Agriculture is responsible for monitoring at the production level. In addition to inspecting sanitary conditions in processing plants, its officers test foods bacteriologically at different phases of production and review results of microbiologic surveillance carried out by the producer. Until 1998, results of these tests were not available for analysis, since the data remained at the local level.

The Ministry of Economy (Consumers Affairs Directorate) conducts 2-year surveys to determine the proportion and level of contamination of foodstuffs considered to be at risk for *L. monocytogenes* contamination and to evaluate the impact of preventive efforts on the frequency of contamination of these foodstuffs. The results of these surveys are available from 1993 on (17,18). The surveys focus on foods considered to be at risk for *L. monocytogenes* contamination because of characteristics favoring its growth; ready-to-eat foods with a long shelf life are of particular interest. These high-risk foods are divided into four categories (meat, dairy, seafood-fish products, and prepared salads), which are again divided into subcategories of food types that are considered comparable with respect to the likelihood of contamination. Dairy products, for example, are divided into subcategories according to whether the product is pasteurized; soft or hard; ripened or fresh; made of cow, goat, or sheep milk; or blue veined or white mold. In the 1993-94 and 1995-96 surveys, products from 27 subcategories of ready-to-eat foods were sampled at the retail level so that the distribution of the subcategories was similar in both surveys. For these ready-to-eat products, we compared the proportion of samples within the four categories of foods that was contaminated in the periods 1993-1994 and 1995-1996.

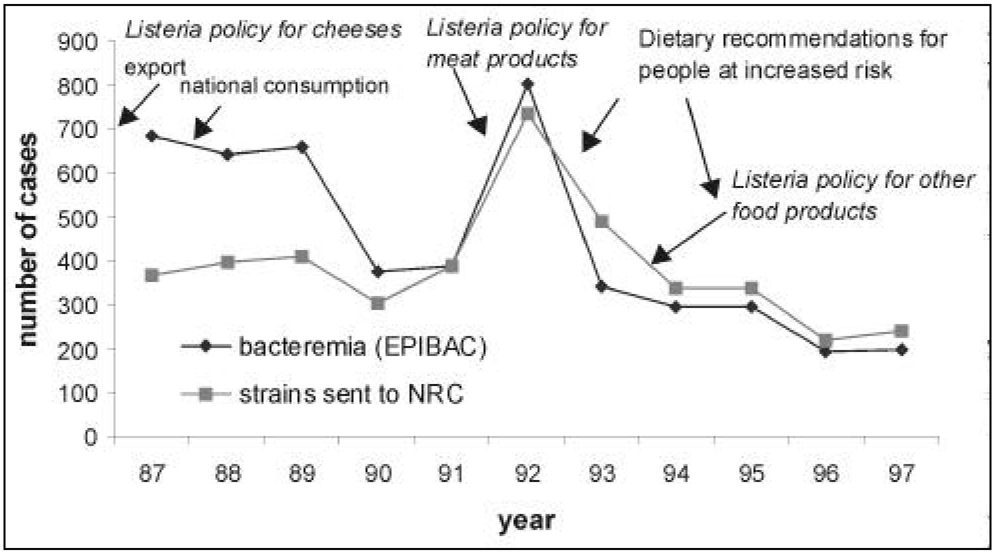

Figure. Annual incidence of *Listeria monocytogenes* bacteremia estimated by the EPIBAC laboratory network, number of isolates received by the National Reference Center, and year of implementation of preventive measures, France, 1987-1997.

### Statistical Analysis

Incidence data were calculated by using the census data estimates from Institut National de la Statistique et des Etudes Economiques (19). For 1987 and 1997, the distribution by category of the identified patients with information on pregnancy status and medical conditions was applied to the total estimated annual incidence to obtain category-specific incidence rates. Changes in incidence by category were evaluated by comparing the category-specific incidence rate in 1987, as estimated by the 1987 hospital laboratory survey, with the category-specific estimated incidence rate in 1997, based on the number of isolates received by NRC.

Since the 1987 survey used a different approach from the 1997 study, we also analyzed the proportional reduction in number of cases by category, from the subset of hospitals that reported cases in both 1997 and 1987. Proportions were compared by chi-square test with Yates correction or Fisher exact test, as appropriate.

### Results

### Incidence of Listeriosis (Figure)

#### EPIBAC

From 1987 to 1993 and from 1994 to 1997, participating hospitals represented 35% and 60%, respectively, of public hospital admissions in medicine departments in France. Participation of regional/university hospitals was slightly but consistently higher than that of the general hospitals: in EPIBAC hospitals, 42% of hospital admissions were in university/regional hospitals; the figure for the whole country is 37.5%. The number of admissions in pediatric departments divided by the number of admissions in all medicine departments (all nonsurgical) was consistently the same (14%) in EPIBAC hospitals and in all hospitals in France.

Using data from the EPIBAC network, we found the annual estimated incidence of bacteremia and meningitis due to *L. monocytogenes* declined substantially, from 12.3 cases for bacteremia and 3.4 cases of meningitis per million population in 1987 to 3.5 cases of bacteremia and 0.9 cases for meningitis per million population in 1997, a reduction of 72% and 73%, respectively. The incidence decreased slightly from 1987 to 1989, showed a clear decrease in 1990, and continued to diminish until 1997, with the exception of 1992, when a large outbreak involving 279 cases occurred in France.

The annual number of cases identified by NRC decreased by 33% from 366 (6.3 cases/million) in 1987 to 242 (4.1 cases per million) in 1997. The trend is similar to that in EPIBAC: a decrease was observed in 1990, followed by a peak in 1992, and then a decrease after 1993. The number of cases identified by the NRC in 1994-1995 was similar to the number identified in 1988-1989, whereas the incidence estimated by EPIBAC shows a 54% reduction over this period.

### Hospital Laboratory Surveys

Overall, the hospitals participating in the hospital surveys represented 71% (1987) and 74% (1988) of all public hospitals beds in France. The participation of regional/university hospitals was higher than that of general hospitals: in 1987 and 1988, the participating regional/university hospitals represented 77% and 83%, respectively, of the regional/university hospital beds in France; the participating general hospitals represented 67% and 70%, respectively, of the general hospital beds in France. The proportion of pregnancy-associated cases was similar in regional/university hospitals (53%) and general hospitals (49%).

Through these surveys, the incidence of listeriosis was estimated in 1987 at 16.7 and in 1988 at 14.9 cases/million population. The surveys show a decrease in incidence of 11% from 1987 to 1988, slightly higher than the 6% decrease observed by EPIBAC over the same period.

Table 1. Incidence and proportion of listeriosis cases by category of patient in 1987 and 1997, France

| Patient categories | Cases estimated per 1,000,000 population | | Percent change | % of cases | |
|---|---|---|---|---|---|
| | 1987[a] | 1997[b] | % | 1987[a] | 1997[b] |
| Pregnancy-associated | 8.5 | 1.3 | -84 | 51 | 24 |
| <65 years of age with no predisposing condition | 1.7 | 0.3 | -82 | 10 | 6 |
| ≥65 years of age without predisposing condition | 1.3 | 0.5 | -62 | 8 | 9 |
| Presence of predisposing condition | 5.3 | 3.3 | -37 | 31 | 61 |
| Total | 16.7 | 5.4 | -68 | 100 | 100 |

[a] Hospital laboratory survey.
[b] National Reference Center

Table 2. Number of listeriosis cases by category of patient in 1987 and 1997, France: comparison of results from the total data set of two surveys and from a subset of hospitals reporting cases in both surveys

| Patient category | All hospitals | | | Subset of hospitals reporting cases in 1987 and 1997 | | |
|---|---|---|---|---|---|---|
| | Cases identified | | | Cases identified | | |
| | 1987[a] | 1997 [b] | Percent change[c] | 1987[a] | 1997[b] | Percent change |
| Pregnancy-associated | 336 | 58 | -83 | 166 | 25 | -85 |
| No predisposing condition; <65 years of age | 66 | 13 | -80 | 36 | 6 | -84 |
| Predisposing condition; ≥65 years of age | 51 | 22 | -57 | 19 | 8 | -58 |
| Predisposing condition | 208 | 148 | -29 | 112 | 85 | -24 |
| Total | 661 | 241 | -64 | 333 | 124 | -63 |

[a]Hospital laboratory survey.
[b]National Reference Center.
[c]The percent change in number of cases has been calculated instead of the percent change in incidence, to facilitate comparison w ith the figures from the subset of hospitals. As a result, the figures on percent change are slightly lower than those in Table 1, which take into account the increase in the French population during the 10-year period.

### Incidence by Category of Patient

The decrease in incidence was most prominent among pregnant women (84%) and persons <65 years of age without predisposing conditions (82%) (Table 1). The decrease was less marked among elderly subjects (≥65 years of age) without predisposing condition (62%) and much lower among persons with a predisposing condition (37%). Analysis of the subset of hospitals that participated in both the 1987 and the 1997 study yielded very similar results as the analysis of the total data set (Table 2). As a result, the relative importance of different categories of patients changed drastically. In 1987, 51% of the cases were pregnancy associated, but in 1997 this category represented 24% (Table 1). Patients with a predisposing condition represented only 31% of all cases in 1987 but accounted for 61% of all cases in 1997.

### Food Monitoring

In 1993-94 and in 1995-96, a total of 5,809 and 6,147 ready-to-eat products were sampled, respectively. Overall, meat products (10.8%) and seafood-fish products (10.4%) were contaminated more frequently than dairy products (4.7%) and prepared salads (4.5%). However, dairy products were more frequently contaminated at higher doses than other products: 1.8% of dairy products were found to be contaminated with ≥100 CFU/g, compared with 0.3%, 0.5%, and 1.1% for salads, seafood/fish products, and meat products, respectively (Table 3).

During 1993-94 and 1995-96, we observed a decrease in the proportion of contaminated products that was greater for products contaminated with ≥100 CFU/g (38%) than for products contaminated with <100 CFU/g (10%) (Table 4). The decrease in the proportion of products with contamination ≥100 CFU/g was 56% for dairy products and 41% for meat products. Foods with levels of contamination <100 CFU/g decreased by 33% for prepared salads and 23% for meat products, but no substantial change was observed for dairy products and seafood-fish products.

### Discussion

The results indicate that the incidence of invasive disease by *L. monocytogenes* decreased substantially from 1987 through 1997. The reduction in incidence was 72% when estimated through the EPIBAC network and 68% when estimated by comparing incidence rates from the 1987 hospital laboratory survey and the NRC in 1997. Since the 1987 survey used a different approach from the 1997 study, the difference in sensitivity of the two approaches may have resulted in or contributed to the reduction observed. However, analysis of the subset of hospitals that participated in both the 1987 and 1997 studies provided comparable data and yielded results very similar to those of the analysis of the total data set. The number of cases identified through the public hospital laboratory surveys also decreased from 1987 to 1988, consistent with the trend observed by the EPIBAC network.

Table 3. Proportion of ready-to-eat foods contaminated with *Listeria monocytogenes* (Lm), by food category, France, 1993-1996

| | Ready-to-eat meat products | Dairy products | Prepared salads | Seafood-fish products |
|---|---|---|---|---|
| Number of samples | 3,283 | 3,541 | 3,166 | 1,966 |
| % of food contaminated by Lm | | | | |
| <100 CFU/g[a] | 9.7 | 2.9 | 4.2 | 9.7 |
| ≥100 CFU/g | 1.1 | 1.8 | 0.3 | 0.5 |
| Total | 10.8 | 4.7 | 4.5 | 10.4 |

[a]Levels of contamination <100 CFU/g but greater than zero.

Table 4. Proportion of ready-to-eat foods contaminated with *Listeria monocytogenes* (Lm) by food category, level of contamination, and period, France, 1993-1996

| | 1993-94 | 1995-96 | Percent change (%) | p[a] |
|---|---|---|---|---|
| **Ready-to-eat meat products** | | | | |
| Number of samples | 1,533 | 1,750 | | |
| % of food contaminated by Lm | | | | |
| <100 CFU/g[b] | 11 | 8.5 | -23 | 0.02 |
| ≥100 CFU/g | 1.6 | 0.7 | -56 | 0.03 |
| Total | 12.6 | 9.2 | -28 | 0.003 |
| **Dairy products** | | | | |
| Number of samples | 1,695 | 1,846 | | |
| % of food contaminated by Lm | | | | |
| <100 CFU/g[b] | 3.2 | 2.7 | -16 | ns |
| ≥100 CFU/g | 2.2 | 1.3 | -41 | 0.03 |
| Total | 5.4 | 4 | -26 | 0.03 |
| **Prepared salads** | | | | |
| Number of samples | 1,740 | 1,426 | | |
| % of food contaminated by Lm | | | | |
| <100 CFU/g[b] | 4.9 | 3.3 | -33 | 0.02 |
| ≥100 CFU/g | 0.3 | 0.3 | 0 | ns |
| Total | 5.2 | 3.6 | -31 | 0.02 |
| **Seafood–fish products** | | | | |
| Number of samples | 841 | 1,125 | | |
| % of food contaminated by Lm | | | | |
| <100 CFU/g[b] | 9.3 | 11.4 | +22 | ns |
| ≥100 CFU/g | 0.7 | 0.5 | -29 | ns |
| **Total** | 10 | 10.3 | +18 | ns |
| Total no. of samples | 5,809 | 6,147 | | |
| % of food contaminated by Lm | | | | |
| <100 CFU/g[b] | 6.6 | 5.9 | -10 | ns |
| ≥100 CFU/g | 1.3 | 0.8 | -38 | 0.007 |
| Total | 7.9 | 6.7 | -16 | 0.008 |

[a]Chi-square test with Yates correction or Fisher exact test.
[b]Levels of contamination <100 CFU/g but greater than zero.

Data on the incidence by category of patient clearly indicate that the largest reduction in incidence occurred in pregnant women and previously healthy adults <65 years of age. A similar observation was made in the United States from 1989 to 1993, where an overall reduction in incidence of 44% was observed (51% in pregnancy-associated cases and 37% in patients >50 years of age), and in England and Wales, where the proportion of pregnancy-associated cases declined from 31%-48% between 1983 and 1989 to 8%-26% between 1990 and 1996 (2,20).

During the period that the decrease in incidence was observed, prevention measures have been progressively introduced by the food industry. Food monitoring data strongly suggest that these measures have successfully reduced the distribution of *L. monocytogenes*-contaminated ready-to-eat foodstuffs. The temporal relationship between the preventive measures, the reduction in *L. monocytogenes*-contaminated foodstuffs, and the decrease in incidence supports a causal relationship. A similar temporal association of reduction in incidence with implementation of prevention measures was observed in the U.S. study (2). The incidence of listeriosis per million population, much higher in 1986 in France (14.7) than in the United States (7.3), was similar in 1997 (France 5.4; USA 4.8) (21-23). Thus, the reduction in rate from 1986 to 1997 was much greater in France.

Dietary recommendations for immunocompromised patients and pregnant women to avoid certain foods may have contributed to the decline in incidence. However, several findings suggest that this contribution is unlikely to have been substantial. First, although dietary recommendations were targeted only at immunocompromised persons, the elderly, and pregnant women—never at previously healthy adults <65 years old—an 83% reduction in incidence was observed in this category. In addition, incidence began to decline in the period 1987 to 1992, when prevention measures at the production level were the only steps taken to prevent listeriosis in France.

Food monitoring data indicate that the reduction in *L. monocytogenes*-contaminated products was greatest for more heavily contaminated products, with an overall decrease of 38% for products contaminated at $\geq$100 CFU/g, compared with 10% for products contaminated at <100 CFU/g. The findings that the decrease in contaminated products was most pronounced for the more heavily contaminated products and that the reduction in incidence was most important among previously healthy adults and pregnancy-associated cases support the hypothesis that these two groups need to be exposed to a higher infectious dose for invasive illness to develop, as demonstrated by dose-response curves (24). This hypothesis is consistent with the finding that, in several outbreaks in France linked to a highly contaminated food, previously healthy adults and pregnancy-associated cases represent a far larger proportion of cases than among sporadic cases of listeriosis (25,26).

In summary, in France, the incidence of invasive disease due to *L. monocytogenes* decreased by an estimated 68% from 1987 to 1997. The decrease started during 1987-1992, when measures by the food industry were the only steps taken in France to prevent listeriosis. The decrease in incidence was particularly important in previously healthy adults, not included in dietary recommendations. These findings suggest that a substantial part of the decrease in illness due to *L. monocytogenes* is related to control measures implemented at the food production level.

Dr. Goulet is an epidemiologist in the Department of Infectious Diseases at the Institut de Veille Sanitaire in France. She coordinates the surveillance and investigation of outbreaks of human listeriosis in France and has studied the epidemiology of human listeriosis since 1984.

### References

1. Schlech WF, Lavigne PM, Bortolusse R, Allen AC, Haldane EV, Wort AJ, et al. Epidemic listeriosis: evidence for transmission by food. N Engl J Med 1983;308:203-6.
2. Tappero JW, Schuchat A, Deaver KA, Mascola L, Wenger JD, for the listeriosis study group. Reduction in the incidence of human listeriosis in the United States: Effectiveness of prevention efforts? JAMA 1995;273:1118-22.
3. Ministère de l'Agriculture, Direction de la Qualité, Service Vétérinaire d'Hygiène Alimentaire. Paris: The Ministry; 1986 April. Note de service DO/SVHA/N86/N°8059.
4. Ministère de l'Agriculture, Direction Générale de l'Alimentation, Service Vétérinaire d'Hygiène Alimentaire. Paris: The Ministry; 1988 Feb. Note de service DG-AL/SVHA/N88/N°8026.
5. Ministère de l'Agriculture, Direction Générale de l'Alimentation, Sous-Direction de l'Hygiène Alimentaire. Paris: The Ministry; 1992 Nov. Note de service DGAL/SDHA/N92/N°8167.
6. Goulet V, Lepoutre A, Rocourt J, Courtieu AL, Dehaumont P, Veit P. Epidémie de listériose en France: Bilan final et résultats de l'enquête épidémiologique. Bulletin Epidémiologique Hebdomadaire 1993;4:13-4.
7. Ministère de l'Agriculture, Direction Générale de l'Alimentation, Sous-Direction de l'Hygiène Alimentaire. Paris: The Ministry; 1992 Sept. Note de service DGAL/SDHA/N92/N°8148.
8. Ministère de l'Economie et des Finances, Direction Générale de la Concurrence, de la Consommation et de la Répression des Fraudes, Bureau de l'Hygiène. Paris: The Ministry; 1992 Dec. Note de Service H2/5882.
9. Ministère de l'Agriculture, Direction Générale de l'Alimentation, Sous-Direction de l'Hygiène Alimentaire. Paris: The Ministry; 1993 Jul. Note de service DGAL/SDHA/N93/N°8108.
10. Jacquet Ch, Saint-Cloment C, Brouille F, Catimel B, Rocourt J. La listériose humaine en France en 1997. Données de Centre National de Référence des *Listeria*. Bulletin Epidémiologique Hebdomadaire 1998;33:142-3.
11. Goulet V, Le Magny F, Rebiere I, Espaze EP. La listériose en France en 1987. Etude rétrospective à partir d'un échantillon d'hôpitaux publics. Bulletin Epidémiologique Hebdomadaire 1989;12:45-6.
12. Goulet V, Mamet J-P, Le Magny F, Rebiere I, Espaze EP. La listériose en France en 1988. Etude rétrospective à partir d'un échantillon d'hôpitaux publics. Bulletin Epidémiologique Hebdomadaire 1990;33:141-2.
13. Infections invasives à *Haemophilus influenzae, Listeria monocytogenes*, méningocoque, pneumocoque, streptocoques A et B en France en 1997. Annual Epidemiological Report. Infectious Diseases Epidemiology in France in 1997. Saint-Maurice, France: Réseau National de Santé Publique; 1999.
14. Jensen A, Frederiksen W, Gerner-Smidt P. Risk factors for listeriosis in Denmark, 1989-1990. Scand J Infect Dis 1994;26:171-8.
15. Espaze EP, Courtieu AL. Rapport du Centre National de Référence des Listeria, 1986. Bulletin Epidémiologique Hebdomadaire 1987;39:15-6.
16. Hook EB, Regal RR. Capture-recapture methods in epidemiology: methods and limitations. Epidemiol Rev 1995;17:243-64.
17. Pierre O, Veit P. Plan de surveillance de la contamination par *Listeria monocytogenes* des aliments distribués. Résultats des plans 1993 et 1994. Bulletin Epidémiologique Hebdomadaire 1996;45:195-7.
18. Ministère de l'Economie, des Finances et de l'Industrie, Direction Générale de la Concurrence, de la Consommation et de la Répresssion des Fraudes. Le plan de surveillance 1993-1996 de la contamination des aliments par *Listeria monocytogenes*. Le Point Sur 1998;9:1-24.

19. Institut National de la Statistique et des Etudes Economiques (France). Estimation de population, évolution 1975-1996. Paris: The Institute; 1997.
20. Listeriosis in England and Wales: 1983 to 1996. Commun Dis Rep CDR 1997;7:95.
21. Goulet V, Brohier S. La listériose en France en 1986: Recensement auprès de laboratoires hospitaliers. Path Biol 1989;37:206-11.
22. Gellin B, Broome C, Bibb W, Weaver R, Gaventa S, Mascola L, et al. The epidemiology of listeriosis in the United States: 1986. Am J Epidemiol 1991;133:392-401.
23. Wallace DJ, Van Gilder T, Shallow S, Fiorentino T, Segler SD, Smith KE, et al. Incidence of foodborne illnesses reported by the foodborne diseases active surveillance network (FoodNet): 1997. J Food Prot 2000;63:807-9.
24. Buchanan R, Lindqvist R. Joint FAO/WHO activities on risk assessment of microbiological hazards in foods. Hazard identification and hazard characterization of *Listeria monocytogenes* in ready-to-eat foods. Preliminary report. MRA 00/01. Geneva: World Health Organization; 2000.
25. Goulet V, Rocourt J, Rebiere I, Jacquet Ch, Moyse C, Dehaumont P, at al. Listeriosis outbreak associated with the consumption of rillettes in France. J Infect Dis 1998;177:155-60.
26. Vaillant V, Maillot E, Charley C, Stainer F. Epidémie de listériose, France Avril-Août 1995. Saint-Maurice, France: Rapport du Réseau National de Santé Publique; 1998. p. 1-58.
